# Vibrotactile Feedback Improves Manual Control of Tilt After Spaceflight

**DOI:** 10.3389/fphys.2018.01850

**Published:** 2018-12-19

**Authors:** Gilles Clément, Millard F. Reschke, Scott J. Wood

**Affiliations:** ^1^Lyon Neuroscience Research Center, Bron, France; ^2^KBRwyle, Houston, TX, United States; ^3^Neuroscience Laboratories, National Aeronautics and Space Administration Johnson Space Center, Houston, TX, United States; ^4^Azusa Pacific University, Azusa, CA, United States

**Keywords:** vestibular system, manual control, vibrotactile feedback, microgravity, subjective vertical

## Abstract

The objectives of this study were to quantify decrements in controlling tilt on astronauts immediately after short-duration spaceflight, and to evaluate vibrotactile feedback of tilt as a potential countermeasure. Eleven subjects were rotated on a variable radius centrifuge (216°/s <20 cm radius) in a darkened room to elicit tilt disturbance in roll (≤± 15°). Nine of these subjects performed a nulling task in the pitch plane (≤±7.5°). Small tactors placed around the torso vibrated at 250 Hz to provide tactile feedback when the body tilt exceeded predetermined levels. The subjects performed closed-loop nulling tasks during random tilt steps with and without this vibrotactile feedback of tilt. There was a significant effect of spaceflight on the performance of the nulling tasks based on root mean square error. Performance returned to baseline levels 1–2 days after landing. Vibrotactile feedback significantly improved performance of nulling tilt during all test sessions. Nulling performance in roll was significantly correlated with performance in pitch. These results indicate that adaptive changes in astronauts’ vestibular processing during spaceflight impair their ability to manually control tilt following transitions between gravitational environments. A simple vibrotactile prosthesis improves their ability to null-out tilt within a limited range of motion disturbances.

## Introduction

We previously compared perceptual and ocular changes in astronauts by tilting them in roll and pitch before and immediately after short-duration spaceflight. The astronauts overestimated tilt and translation as a result of their adaptation to weightlessness (Clément and Wood, 2014; Clarke and Schönfeld, 2015). When a spacecraft is accelerating or decelerating, the automated processes for determining the position of the horizon are not very accurate and the pilot must manually correct the tilt of the vehicle. During future exploration mission to Mars where dust clouds are common, it will be critical that the astronaut pilots estimate the amplitude of the vehicle tilt and manually level the vehicle in the absence of any visual cues. Changes in perceived body position caused by adaption to weightlessness could impair an astronaut’s ability to manually correct the position of spacecraft and this could have dire consequences (Paloski et al., 2008). To further define this risk of impaired performance, we assessed how spaceflight affects astronauts’ abilities to perform a manual-nulling task during passive body tilt in pitch and roll.

Although all the Space Shuttle landings were successful, the pilots’ landing performances varied. Landing speeds of the Space Shuttle varied considerably: 20% of the first 100 touchdowns were faster than acceptable, and six were so fast they risked damaging the landing gear tires (Moore et al., 2008). In addition, Clark and Bacal (2008) noted that the two fastest landings were linked to the pilot’s momentary spatial disorientation. The degree of neurovestibular dysfunction in the astronauts within several hours of landing (as measured by subjective symptoms, spatial disorientation, impairment in locomotion and coordination of movements, and functional motor performance) was negatively correlated with their performance navigating the spacecraft during the landing procedure.

Previous investigations assessed how nine astronauts manually controlled lateral translations during linear acceleration 2 days after they returned from 10 days Spacelab missions (Arrott and Young, 1986; Arrott et al., 1990). Astronauts were seated in a cabin that was mounted perpendicular to a sled and they were tested while the sled moved in a random appearing velocity profile made of 12 sinusoids added together. Subjects were asked to null out the pseudo-random disturbance of lateral motion using a joystick that controlled the velocity of the sled. On landing day, most astronauts performed this task in the dark better than they did before the mission. No spaceflight-induced change in performance was observed when they completed the same task with visual cues. Unfortunately, the astronauts did not report any sense of roll tilt during lateral translation on the sled. After the mission, the astronauts’ performance appeared to improve relative to preflight performance during the higher frequencies of the profile used, which were more likely to generate a sense of translation (Wood, 2002).

Merfeld (1996) measured how two astronauts controlled roll tilt after the 14 days Space Life Science-2 Spacelab mission. Subjects sat on a motion platform that tilted from 0.4 to 2.4° in a pseudo-random sinusoidal profile. On landing day, the two subjects’ ability to maintain an upright orientation was greatly impaired compared to their ability before the flight. Clark et al. (2015a) tested a similar manual control task while rotating subjects to 1.5 and 2 Gz in a ground-based centrifuge. Performance of the roll motion-nulling task in darkness degraded during the first trials, but subjects improved their performance over time (Clark et al., 2015b).

Further tests were conducted on the Spacelab astronauts during larger amplitude roll tilts, which correspond to tilts pilots experience when they are flying a spacecraft. Studies using both actual body tilt and centrifugation have shown that immediately after spaceflight the astronauts overestimated the tilt of their body in roll relative to gravity (Reschke and Parker, 1987; Glasauer and Mittelstaedt, 1998; Clément et al., 2001; Clément and Wood, 2013, 2014). We found that subjective estimates of the amplitude of static body tilt in pitch 2 days after landing were unchanged from preflight values (Clément and Wood, 2014). Ocular counter-rolling and counter-pitching, i.e., the compensatory eye movement in response to head roll and pitch tilt, respectively, were not significantly altered (Clément et al., 2007).

The primary goal of the present study was to quantify the decrements in controlling both roll and pitch tilt in a larger group of astronauts immediately after spaceflight. The secondary goal was to test the efficacy of a countermeasure for mitigating these decrements. Vibrotactile feedback (also referred to as haptic feedback) improves balance by using the sense of touch to substitute for, or augment, the sense of sight and balance (Shull and Damian, 2015; Sienko et al., 2017). Vibrotactor arrays placed around the waist of individuals with vestibular deficits can help them reduce tilts of their heads and displacements of their center of pressure while they are standing with their eyes closed (Kentala et al., 2003; Wall and Kentala, 2005; Wall, 2010). The U.S. Navy developed a tactile situation awareness system (TSAS) to cue pilots on the orientation of their aircraft relative to gravity during aerial navigation and combat (Rupert, 2000). The vibration of tactors distributed on the subjects’ torso cued them to move in the opposite direction of vibration, and the location of vibrating tactor indicated the degree of desired correction.

More recently, Sienko et al. (2008) found that four tactors spaced evenly around the waist were as effective for correcting posture sway as an array of 48 tactors (3 rows by 16 columns) placed around the waist. In the present study we used a belt with pairs of tactors in each direction of tilt: two tactors were aligned on the front and on the back of the torso during pitch tilt tests, and two were aligned on the right and on the left of the torso during roll tilt tests. We activated the tactors when the chair offset exceeded preset values. We compared tilt nulling performance in pitch and roll before and after spaceflight, and with and without this tactile feedback.

## Materials and Methods

### Subjects

Eleven crewmembers (10 males, 1 female; age 42–55 years, mean 49 years) participated in this experiment. Each crewmember flew on one of eight Space Shuttle missions lasting 11–15 days. All subjects had normal neurological function, as evaluated during the NASA astronaut selection process and subsequent annual medical examination. This study was carried out in accordance with the recommendations of NASA Johnson Space Center Institutional Review Board. The protocol was approved by NASA Johnson Space Center Institutional Review Board. All subjects gave written informed consent in accordance with the Declaration of Helsinki.

All subjects were tested three times before the mission at the Neuroscience Laboratory of the NASA Johnson Space Center (JSC) in Houston at approximately launch minus (L-) 120 days, L-90 days, and L-60 days. The initial post-flight test for the roll tilt stimuli was typically performed between 1 and 4 h after return to Earth (R+0 day) tests at the Space Shuttle landing sites at the Kennedy Space Center or the Dryden (now Armstrong) Space Research Center. Testing was delayed until return to NASA JSC on five subjects due to motion sensitivity or equipment malfunction. Nine subjects participated in the pitch tilt stimuli immediately upon return to NASA JSC during the day following the return to Earth (R+1). Return to baseline performance was monitored with continued post-flight testing at NASA JSC on R+2 and R+4 days.

### Roll Tilt

**FIGURE 1:**
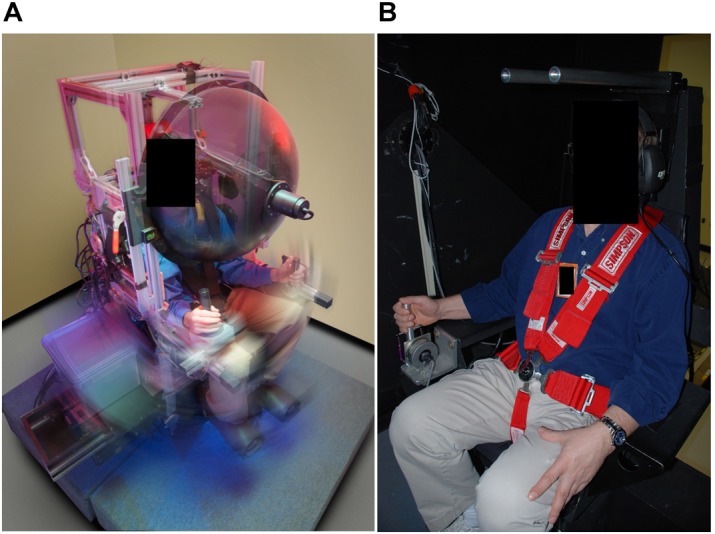
**(A)** Roll tilt The variable radius centrifuge consisted of a servo-controlled rotator with a small linear track to provide dynamic translation of the chair during constant velocity rotation. Controlling the joystick with their right hand, subjects attempted to keep the chair in the center of the lateral translation stage (maintain a perceived upright orientation). **(B)** Pitch tilt Subjects sat in a chair that tilted in pitch about the interaural axis while they performed a closed-loop nulling task in which they used a joystick to null out perceived tilt motion. Photo credit: NASA.

A variable radius centrifuge was used to generate a centripetal acceleration along the subjects’ interaural axis, which elicited a perception of tilt (somatogravic illusion) in roll without concordant roll cues from the semicircular canals canal or visual cues (Clark and Graybiel, 1966). Subjects were restrained in a chair that was mounted on a small translation stage fixed to rotator that turned about the vertical axis in a light tight enclosure (Figure 1A). The restraint system incorporated straps and padding at shoulders, mid-torso, waist, thighs, and feet. Support was provided by moldable Vac-Pacs (Olympic Medical, Seattle, WA, United States) that helped immobilize the body and distribute the pressure uniformly during tilt. A head restraint with adjustable foam pads provided even pressure and head stability relative to the chair. The height and fore-aft position of the head restraint was adjusted to accommodate different subjects while restraining their head in a naturally upright orientation.

The roll stimuli involved an integrated protocol with Clarke and Schönfeld (2015) where the centrifuge was either slowly accelerated (3°/s^2^) to a constant velocity of 216°/s or decelerated if a unilateral eccentric rotation paradigm was performed first. In either case, subjects rotated for 60 s at constant velocity to allow the post-rotatory response of semicircular canals to decay. After the subjects no longer sensed the rotation, the chair was displaced using the translation stage by ±6.1, ± 12.2, and ±18.5 cm in a random order for 5 s at each position, corresponding to a static roll-tilt of the gravitoinertial acceleration vector at ±5°, ± 10°, and ±15°, respectively. Throughout the random displacements, subjects were instructed to use a chair-mounted joystick to control the chair’s translation motion and orient themselves to what they perceived to be an upright orientation.

### Pitch Tilt

The subjects were restrained in a tilt chair that was mounted inside a light-tight enclosure. The chair could rotate in pitch about the horizontal axis by means of a direct drive servomotor and a pivoting yoke assembly. The subjects were restrained in the chair with straps and padding around their shoulders, mid-torso, and waist. The chair height was adjusted to align the subject’s head inter-aural axis with the tilt axis, and their head was restrained in an upright orientation. The chair was then tilted in pitch at ±2.5°, ± 5°, and ± 7.5° in a random order for 5 s at each angle. As with roll, subjects were instructed to use a chair-mounted joystick to orient the chair to what they perceived as upright (Figure 1B), i.e., null out the tilt disturbances. In both the tilt chair and the centrifuge, noise-canceling headphones were used for two-way audio communications and for suppressing any auditory cues of spatial orientation.

### Vibrotactile Feedback

Small (0.3 inch in diameter) electromechanical vibrators (C2 model, Engineering Acoustics Inc., Winter Park, FL, United States) provided vibrotactile feedback regarding the direction and amplitude of tilt. The vibration was similar to vibration mode on cell phones (Wood et al., 2009). Before getting into the chair, subjects donned a belt that had four of these tactors. Two tactors were positioned vertically on the front and two on the back of the torso during pitch tilt; two tactors were positioned vertically on the right and two on the left of the torso during roll tilt. Information on body orientation was derived from encoders mounted to the drive axes. Tactors provided a steady pulse rate (250 Hz) that indicated both direction and magnitude of tilt. The lower tactor was activated when body tilt reached 2° relative to gravity; the upper tactor was activated when body tilt reached 4° relative to gravity; both the lower and upper tactors were activated when body tilt reached 6° relative to gravity. Subjects were trained to use the vibrotactile feedback of tilt at the beginning of the first two preflight sessions. To minimize any learning effects, only the last preflight session was compared with the post-flight measures. The order of trials with and without vibrotactile feedback was also counterbalanced across subjects.

### Data Analysis

Nulling task performance was derived from the Root Mean Squared (RMS) error in degrees across each entire trial. Thus, lower RMS error represented improved nulling performance relative to higher RMS error. RMS error was obtained from each pitch and roll session for both nulling with and without vibrotactile performance. Based on Shapiro–Wilk tests, the data were not consistently normally distributed. Therefore, only non-parametric statistical tests were utilized.

For both pitch and roll nulling performance, the effect of spaceflight was evaluated with a related samples Friedman’s Analysis of Variance by ranks using preflight, R+0/1, R+2, and R+4 measurements. The effect of vibrotactile feedback on nulling performance was based on a related samples Wilcoxon Signed Rank test. Finally, the relationship between nulling performance in pitch and roll was evaluated with the Spearman’s rho. A the critical statistic of *p* < 0.05 was used for all analytical testing.

The first and second preflight sessions were considered familiarization training for the initial exposures to the nulling task. Although there was no significant difference in RMS measures across the three preflight sessions for roll and pitch planes with or without vibrotactile feedback (Friedman’s ANOVA), we felt that the latest preflight session was the most appropriate baseline measure since it would minimize any learning or recency effects. There did appear to be a trend of continued improvement during the later post-flight period, presumably due to learning effect with more frequent testing.

## Results

**FIGURE 2:**
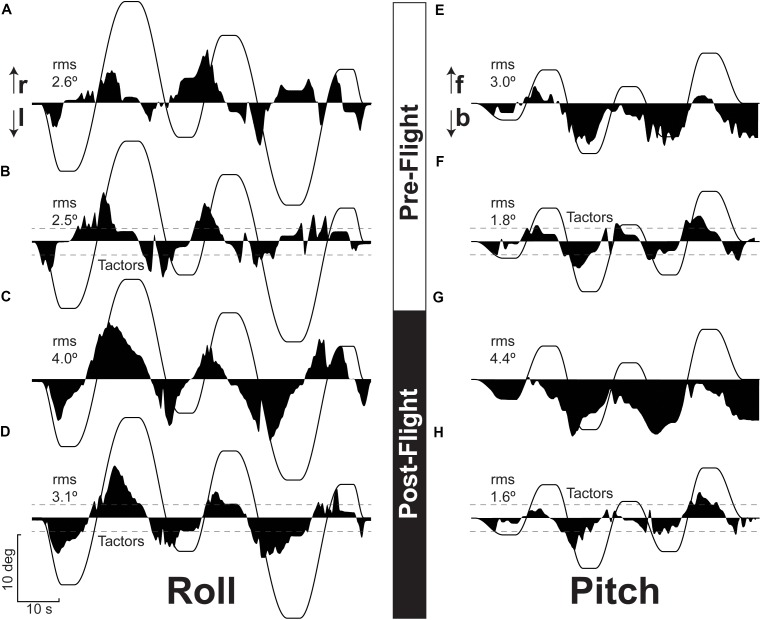
Typical recordings of residual chair tilt after nulling (dark area) in response to imposed chair tilt (thin line) in roll (left side) and pitch (right side) before (top half) and after (bottom half) spaceflight without and with tactile tactile feedback. Performance without vibrotactile feedback is shown in **(A,C,E,G)**. Performance during the same sessions with vibrotactile feedback is shown in **(B,D,F,H)**. The dashed lines on these traces indicate the lowest threshold at which vibrotactile feedback was provided. The RMS error value for each test in also reported. r, right, l, left; f, forward; b, backward.

Figure 2 illustrates the typical nulling performance during roll and pitch tilt for both preflight and post-flight tests. As seen in this figure, the deviations in roll were typically symmetrical while the deviations in pitch tended to be asymmetrical with deviations more prevalent in the backward direction. The nulling performance was generally better in the roll plane compared to the pitch plane. A comparison of Figure 2A with Figure 2C and Figure 2E with Figure 2G exemplifies that larger RMS errors were observed post-flight relative to preflight.

**FIGURE 3:**
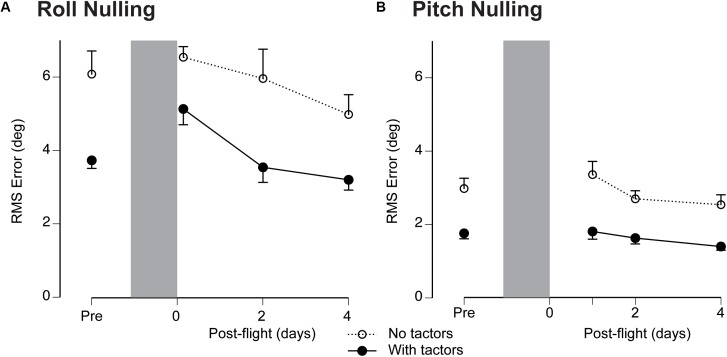
RMS error values averaged for all subjects (±SE) for the nulling tasks during roll tilt **(A)** and pitch tilt **(B)** before (Pre) and after spaceflight without (open symbols) and with (filled symbols) the tactors. Shaded areas represent the flight (not at scale).

Based on the Friedman’s ANOVA, the effect of spaceflight was significant for both roll (*p* < 0.001) and pitch (*p* = 0.047). There was a significant difference in RMS error during roll tilt between preflight and R+0 (Wilcoxon signed rank, *p* = 0.017). RMS error returned to baseline values for both pitch and roll tilt by R+2 days (Figure 3).

**FIGURE 4:**
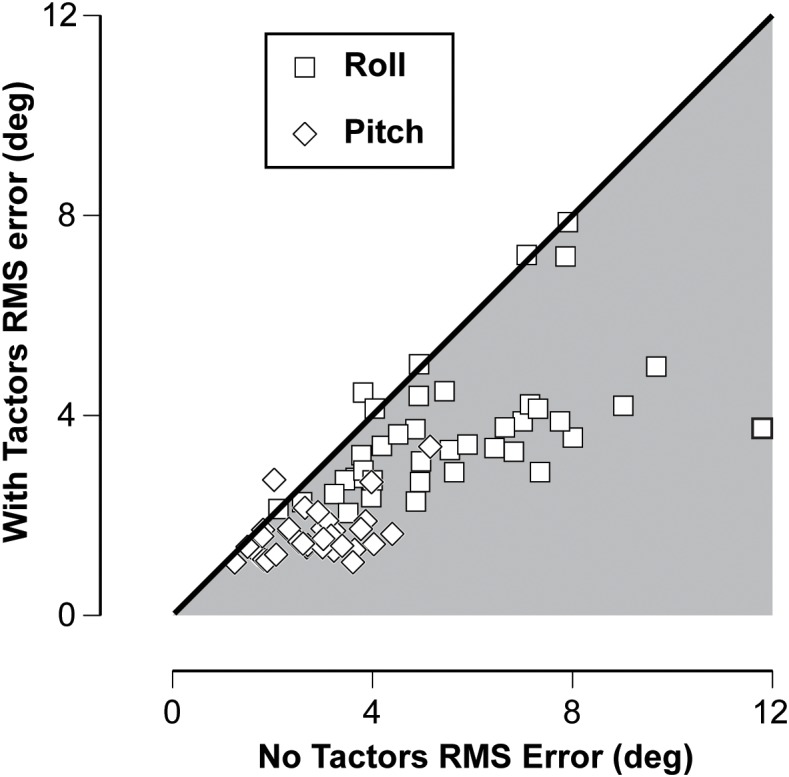
Plot of RMS error values with and without vibrotactile feedback in both the roll and pitch tilt and across all test days for each of the 11 subjects. The unity line illustrates when performance is the same with and without vibrotactile feedback, while the shaded region below this line indicates when performance is improved (RMS error is reduced) with vibrotactile feedback.

The effectiveness of vibrotactile feedback to reduce RMS error both preflight and post-flight is illustrated in Figure 3 by comparing performance without and with vibrotactile feedback. Using Wilcoxon signed rank, the difference in RMS error without and with vibrotactile feedback was significantly reduced during both roll and pitch tilt (*p* < 0.001). The consistency of this improved performance is further illustrated in Figure 4 by plotting the nulling performance with and without vibrotactile performance for each session during roll and pitch tilt. The vast majority of points falls in the shaded region below the unity line, which illustrates that nulling performance was consistently greater with vibrotactile feedback.

**FIGURE 5:**
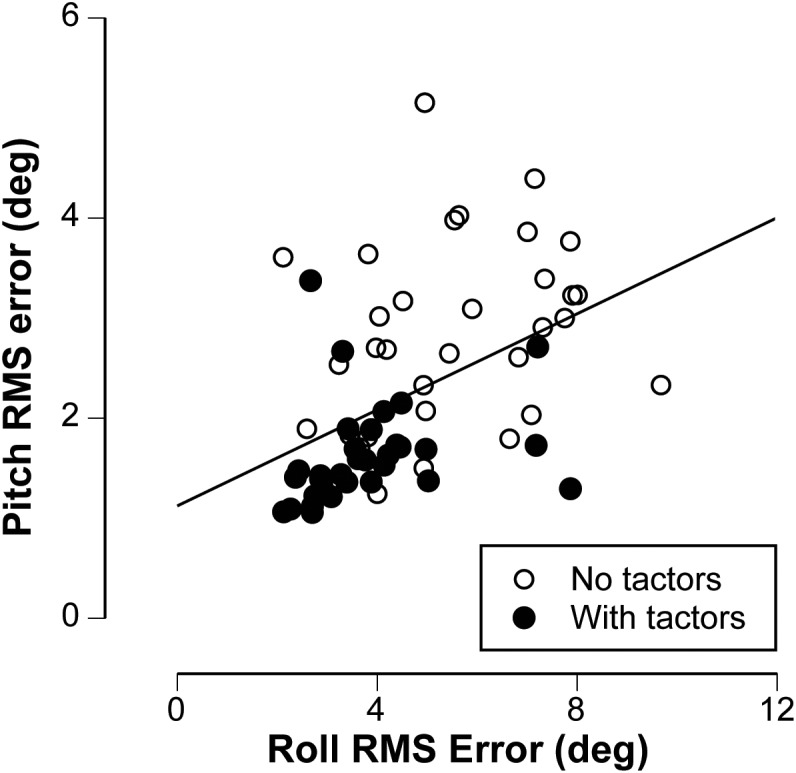
Plot of the relationship between the nulling performance in pitch vs. the nulling performance in roll, including trials without (open symbols) and with (filled symbols) vibrotactile feedback. The line represents best linear fit, characterized by Spearman’s correlation (rho = 0.496, *p* < 0.001).

The larger RMS errors observed during the roll stimuli (compare Figure 3A and Figure 3B) are likely attributable to the greater tilt angles in roll. However, differences in vestibular cues between these two stimuli may also explain the larger errors in roll. The roll stimuli elicited tilt disturbances using centripetal accelerations during variable radius centrifugation in contrast to the concordant otolith and canal cues elicited during pitch stimuli. Nevertheless, it is interesting to evaluate the individual performances across both stimuli. Figure 5 illustrates that there was a significant correlation between an individual’s performance in roll and pitch, including trials during which vibrotactile feedback was utilized. The Spearman’s rho was 0.496 (*p* < 0.001), suggesting that better performers in roll also tended to be better performers in pitch.

## Discussion

The results of the present study agree with those obtained after the Space Life Sciences-2 mission (Merfeld, 1996): astronauts returning from spaceflight missions lasting from 11 to 15 days have difficulties controlling roll tilt in the absence of visual cues. By contrast, their control of pitch tilt is less affected by spaceflight. This difference is in agreement with tilt perception reports that showed astronauts overestimated the amplitude of roll tilt on R+0, but their perception of the amplitude of pitch tilt on R+1 was the same as preflight (Clément and Wood, 2014). Moreover, the improvements in performance using vibrotactile feedback suggests that the astronauts’ motor function, i.e., their manipulation of the joystick, was unaffected by exposure to weightlessness.

When trying to null-out an overestimated tilt in roll immediately after landing, the subjects presumably overshoot and this induces a tilt in the opposite direction and therefore generates more instability. These effects, however, are no longer present on R+2, suggesting that the deficits are due to re-adaptation to gravity, which persist throughout the early post-flight period. One limitation in our study was that the first test using the tilt chair took place at R+1, so it is possible that impairment in controlling pitch present at R+0 had recovered in part by R+1. Nevertheless, we demonstrated a significant effect of spaceflight for both pitch and roll tilt.

One possible interpretation for the impaired control of roll tilt after spaceflight is that perception of tilt in darkness becomes useless in weightlessness because body tilt in space will not result in a fall. The otoliths in the inner ear sense both head translation and head tilt relative to gravity. In weightlessness, head tilt no longer stimulates the otoliths, but they are still stimulated by head translation. Researchers have therefore proposed that during adaptation to weightlessness, the brain interprets all otolith output as translation only, and that this interpretation persists during the early post-flight period (Young et al., 1984; Parker et al., 1985). Other authors have suggested that in weightlessness some neural processes that integrate sensory input might use rotational cues for interpreting ambiguous gravitoinertial signals via internal models (Merfeld et al., 1999; Angelaki and Dickman, 2003; Merfeld, 2003; Angelaki et al., 2004; Zupan and Merfeld, 2005; Clark et al., 2015c).

The hypothesis of multi-sensory convergence suggests that in weightlessness, the brain switches from detecting gravity solely based on signals from the otolith organs to including signals from the semicircular canals (Angelaki and Dickman, 2003; Angelaki et al., 2004). In ground-based studies, Angelaki et al. (1999) used monkeys to show the importance of integrating multi-sensory information to discriminate tilt from translation. The monkeys moved their eyes horizontally to compensate for translation at 0.5 Hz, but their eyes moved only slightly during pure roll tilt. However, when their semicircular canals were plugged, the monkeys moved their eyes horizontally during all linear acceleration, regardless of whether the acceleration resulted from translation or tilt. This result is very similar to how the human eye moves in response to constant velocity rotation >0.3 Hz in an off-vertical axis (Wood, 2002). In the present study the semicircular canals were not stimulated during the roll tilt induced by centrifugation, whereas they were stimulated when the chair was tilted in pitch. The origin of the impairment in nulling performance in roll could be intra-vestibular conflict during centrifugation. However, Merfeld (1996) observed impairment in nulling performance when astronauts were tilted in roll relative to the gravity after spaceflight, which stimulated both the semicircular canals and the otoliths.

The other notable finding from the present study is that a simple vibrotactile sensory aid improves control of tilt when attempting to maintain an upright orientation within a limited tilt range. Previous tests have shown that a TSAS is a promising tool for reducing spatial disorientation in unusual acceleration environments (Rupert, 2000) when sensory cues are limited and sensorimotor function is compromised. Similar technology has been used to aid orientation in aeronautic and space environment (Rochlis and Newman, 2000; Van Erp et al., 2002) and to control balance in vestibular-compromised patients.

A study of one astronaut on board the International Space Station showed that localized vibration on the torso to indicate “down” made orienting in weightlessness faster, better, and easier (Van Erp and Van Veen, 2006). The effectiveness of this tactile aid increased over the first 7 days of staying in microgravity while the relative contribution of visual information to spatial orientation decreased over the same period.

In the recent years, vibrotactile feedback has been used successfully in aviation and to improve rehabilitation of individuals with balance disorders (Rupert, 2000; Wall, 2010). Tactile displays can help individuals to learn and change the limits of their stability while they stand or walk and to “tune” the motion inputs from their extra-vestibular system, which helps them improve their postural control. Wall and Kentala (2005) demonstrated that patients with vestibular dysfunction had improvements in postural performance when using tactile displays that record anterior-posterior motion at their waist. Vibrotactile feedback of tilt may also help the elderly or individuals with injuries that cause them to struggle with acute and chronic imbalance. Balance and vestibular rehabilitation therapy could be initiated in a laboratory setting, then the patients could supplement the clinical training at home using another version of the feedback device, and they could continuously wear a portable version to help prevent falls (Wall, 2010).

Since these effects may be attributable to vestibular (primarily otolith) adaptation to spaceflight, one would expect the differences to be larger following long duration spaceflight. Unfortunately, crewmembers now return from the International Space Station on the Soyuz, which lands in the Kazakhstan desert, and the earliest opportunity to perform post-flight measurements in a laboratory is >22 h after landing. Once the NASA Multipurpose Crew Vehicle or the Space-X Dragon spacecraft are operational, it may be possible to test crewmembers of these vehicles sooner after landing, as it was the case with the Space Shuttle crewmembers.

## Conclusion

The results of this study demonstrate that a simple belt using two tactors on each side improved the performance of a nulling task during all the sessions. An even simpler system that uses only one tactor on each side, cycling from a slow pulse rate to a steady pulse to indicate both the direction and magnitude of tilt, is currently being tested on astronauts returning from long-duration spaceflight on board the International Space Station. Our current understanding of the risk of impaired control of a spacecraft due to vestibular alterations associated with spaceflight is limited. The pilots’ landing performance has been less than desired for both the Space Shuttle and the lunar lander during the Apollo program. Because of the extent physiological adaptation to weightlessness plays in these performance decrements, we can anticipate that the risk of failure will become much greater after a 6 months outbound exploration trip (without artificial gravity) than after a Space Shuttle mission lasting 1–2 weeks. Moreover, the effects of transition from weightlessness to Mars gravity rather than Earth gravity (0.38 g vs. 1 g) is unknown. Tactile feedback, as well as other sensorial countermeasures, including visual, vestibular, auditory, and multisensory displays (Paillard et al., 2014), could potentially mitigate these risks.

## Author Contributions

GC, MR, and SW contributed to study design, data collection, data analysis, and manuscript. All authors reviewed the manuscript.

## Conflict of Interest Statement

The authors declare that the research was conducted in the absence of any commercial or financial relationships that could be construed as a potential conflict of interest.
